# Impact of Polymer Binders on the Structure of Highly Filled Zirconia Feedstocks

**DOI:** 10.3390/polym12102247

**Published:** 2020-09-29

**Authors:** Claire Delaroa, René Fulchiron, Eric Lintingre, Zoé Buniazet, Philippe Cassagnau

**Affiliations:** 1 Ingénierie des Matériaux Polymères, Univ Lyon, Université Lyon 1, CNRS UMR 5223, 15 Boulevard Latarjet, 69622 Villeurbanne CEDEX, France; Claire.Delaroa@saint-gobain.com (C.D.); rene.fulchiron@univ-lyon1.fr (R.F.); 2 Saint-Gobain CREE, Grains et Poudres, 550 Avenue Alphonse Jauffret, BP 20224, 84306 Cavaillon, France; Eric.Lintingre@saint-gobain.com (E.L.); Zoe.Buniazet@saint-gobain.com (Z.B.)

**Keywords:** polymer binder, zirconia feedstocks, ceramic density

## Abstract

The impact of polypropylene and high-density polyethylene backbone binders on the structure of organic matrix, feedstock, and ceramic parts is investigated in terms of morphology in this paper. The miscibility of wax with polyethylene and polypropylene is investigated in the molten state via a rheological study, revealing wax full miscibility with high-density polyethylene and restricted miscibility with polypropylene. Mercury porosimetry measurements realized after wax extraction allow the characterization of wax dispersion in both neat organic blends and zirconia filled feedstocks. Miscibility differences in the molten state highly impact wax dispersion in backbone polymers after cooling: wax is preferentially located in polyethylene phase, while it is easily segregated from polypropylene phase, leading to the creation of large cracks during solvent debinding. The use of a polyethylene/polypropylene ratio higher than 70/30 hinders wax segregation and favors its homogeneous dispersion in organic binder. As zirconia is added to organic blends containing polyethylene, polypropylene, and wax, the pore size distribution created by wax extraction is shifted towards smaller pores. Above zirconia percolation at 40 vol%, the pore size distribution becomes sharp attesting of wax homogeneous dispersion. As the PP content in the organic binder decreases from 100% to 0%, the pore size distribution is reduced of 30%, leading to higher densification ability. In order to ensure a maximal densification of the final ceramic, polyethylene/polypropylene ratios with a minimum content of 70% of high-density polyethylene should be employed.

## 1. Introduction

**Figure 1:**
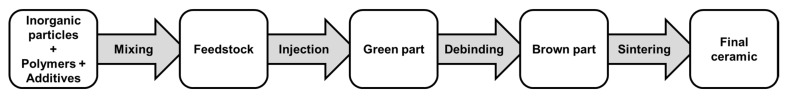
Ceramic injection molding processing steps.

Ceramic injection molding (CIM) allows the production of complex ceramic parts via several processing steps presented in Figure 1. First, high proportions (40–70 vol%) of ceramic fillers, such as zirconia or alumina, are dispersed under high shear rate in an organic binder to create a blend called feedstock. The organic matrix is usually composed of a low molar mass binder, commonly waxes, providing flow properties enabling injection molding, and one or multiple high molar mass polymers, called backbone binders, which develop feedstock mechanical properties. Backbone binders currently used in CIM process are polyolefins (i.e., polypropylene (PP), low-density polyethylene (LDPE), and high-density polyethylene (HDPE)), polyethylene glycol (PEG), polystyrene (PS), ethylene-vinyl acetate (EVA), or poly(methyl methacrylate) (PMMA) [1,2]. As fine ceramic powders used in CIM industry present high aggregation ability, compatibilizers such as stearic or oleic acid are usually added to control fillers dispersion state [3,4,5,6,7].

The produced feedstock is injected in a mold under high injection pressure to create compact green parts. Debinding steps are then employed to extract the organic matrix, which constitutes 30 vol% to 60 vol% of the part. This processing step is critical and the selection of debinding routes has been extensively reviewed in the literature [8,9,10]. In order to favor shape retention, and to prevent defect creation, low molar mass binder is first removed via solvent extraction. Subsequently, the remaining organic contents are thermally degraded to obtain a fragile powder structure called brown part [11]. The latter is sintered to create a dense final ceramic.

Actually, technical ceramics such as doped zirconia require high processing quality to achieve full densification and the expected high mechanical properties. However, critical defects and distortions can be grown at each step of the CIM process, due to unsuitable process or formula parameters [11,12,13]. The latter is responsible for poor dimensional control and low mechanical performances, characterized, for example, by a significant decrease of Weibull modulus, resulting in poor quality parts. In fact, every inhomogeneity created during the process cannot be removed and may transform into a macroscopic defect that may induce critical failure of the final ceramic [14,15,16].

Due to complex processing and multicomponent formulas, numerous parameters might modify feedstock behavior and structure. The influence of processing parameters on defect creation has been extensively reviewed in the open literature. Structural evolutions of the sample during debinding and sintering steps have been monitored by porosimetry [17,18,19], imaging [15,20], and dimensional studies [10,21], in order to understand phenomena involved. Feedstock rheological and mechanical behaviors, which master its processing ability, have been analyzed to predict injection molding behavior [22]. Those properties are strongly influenced by the characteristics of the polymer binder, fillers, and additives, as well as their proportions and interactions [23,24,25].

Even though many feedstock formulas have been developed and industrialized, only few studies focused on the impact of organic matrix formula on feedstock structure. Concerning low-molar mass selection, Hsu et al. studied the impact of wax characteristics on LDPE-based feedstock homogeneity and sintering behavior [26]. Karatas et al. investigated injection ability of feedstock composed of HDPE and various waxes namely paraffin, carnauba, and bee’s waxes [27]. They concluded that the use of paraffin wax offered more appropriate flow properties for the CIM process.

On the other side, the critical role of backbone binder has been attested during the whole process by rheological measurements [23,28] and shape retention studies on debinded and sintered parts [21,29,30]. However, few relationships between feedstock structure and backbone binder selection have been really made.

Setasuwon et al. employed two LDPEs presenting different molar masses as backbone binder and observed no clear impact of molar mass on debinding and sintering behaviors of feedstocks [31].

Kim et al. employed EVA or PE wax as minor binders in paraffin wax based feedstocks [32]. Using mercury porosimetry during wick-debinding, they highlighted a significant modification of porosity evolution. The latter could be relative to a modification in binder affinity; however, the behavior of neat polymer blends was not studied.

Wen et al. employed EVA, PP, LDPE, HDPE, and their blends as backbone binders in feedstocks containing paraffin wax as plasticizer [33]. Using scanning electron microscopy and deviation of density, they concluded that feedstocks composed of multiple backbone binders, such as LDPE/HDPE or PP/HDPE, present the more homogenous structure after mixing, leading to the highest densification ability. They supposed it resulted from synergistic effects of multi-polymer blends; however, the behavior of neat polymer binders was not studied.

Moreover, the impact of low-molar mass and backbone binder ratio on injection ability, green microstructure, and sintering ability has also been reviewed. Wen et al. studied PE-paraffin wax based feedstock and concluded that binders containing 70% of wax presented the best mixing behavior, the sharper pore size distribution after thermal debinding, resulting in the highest ceramic density [34].

However, Tseng and Hsu observed the creation of cracking defects during thermal debinding of PVA-paraffin wax based feedstocks when the content of wax reaches 60% of the organic binder [35]. As the wax content increased, the mechanical properties of feedstock were weakened and became insufficient to withstand internal pressure built up by degradation products. By using a powder-bed favoring the evacuation of molten binder, the creation of defect was avoided. Liu and Tseng also showed that pore size distribution obtained in PVA-paraffin wax-based feedstock after thermal debinding was shifted towards smaller pores when the wax content is reduced, due to enhanced fillers dispersion [6].

Since the 1980s, a number of studies have been conducted to understand and improve CIM process at the industrial scale, leading to the development of many feedstock formulas. However, studies related to the impact of backbone polymer selection on parts structure and properties are scarce. Moreover, none of the existing publications investigated the morphology of both neat and filled polymer blends in order to draw conclusions about the importance of the affinity of backbone and low-molar mass binders.

In this paper, the impact of PP and HDPE backbone binders on the structure of organic matrix, feedstock, and ceramic parts is investigated in terms of morphology. In order to have a good insight of the structure of such highly loaded systems, wax dispersion in both neat organic blends and zirconia filled feedstock is studied and compared depending on the selected backbone polymers. Final ceramic quality is also reviewed to deeply understand the link between backbone binder selection, organic matrix morphology, feedstock structure, and final product densification ability.

## 2. Experimental

### 2.1. Materials

Yttria-stabilized Zirconia powder (CY3Z-RA, Saint-Gobain ZirPro, Handan, China) presenting a d_50_ of 0.3 µm and a specific surface area of 7 m^2^/g was used as filler. Multicomponent organic binders composed of high-density polyethylene (HDPE), polypropylene (PP), and paraffin wax (56 wt%) were used. These binders have been selected based on their injection ability and degradation behaviors. Different weight ratios HDPE/PP between polyethylene and polypropylene were investigated: 0/100, 70/30, 50/50, 30/70, and 100/0. Solid content of the blends varied from 0 vol% to 50 vol%, and fully loaded blends are mentioned as feedstocks. In order to favor fillers dispersion, 0.55 wt% of stearic acid was added, relative to the filler amount. Using contact angle and rheological measurements in previous works, Auscher et al. demonstrated that stearic acid can be chemically bonded to zirconia particles surface, reducing interparticle interactions and improving fillers dispersion state [3,24]. The main characteristics of the organic components are presented in Table 1.

### 2.2. Processing

**Figure 2:**
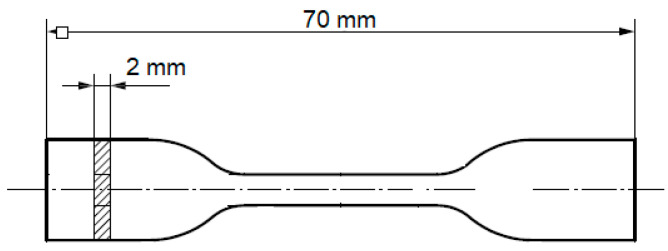
Injected dog bone sample.

Zirconia, stearic acid, and organic binders were mixed for 15 min at 180 °C in an internal mixer (HAAKE Rheomix 600, ThermoFisher, Waltham, MA, US) before being injected (Babyplast 10T, Molteno, Italy) at 170 °C into dog bone samples, presented in Figure 2. Waxes were extracted in an isopropanol bath at 70 °C for 24 h and allowed to dry at room temperature.

Systematic weight tracking of the samples before and after solvent extraction was realized to ensure a complete extraction. As the mass loss of every sample was at least 96 wt% of the wax weight introduced in the blends, we concluded that wax network was highly continuous in the whole sample. Its extraction created an open-pores network characteristic of the sample structure [20]. The latter reflects binder–binder and particle–binder interactions developed in each blend and can be characterized by porosimetry.

**Figure 3:**
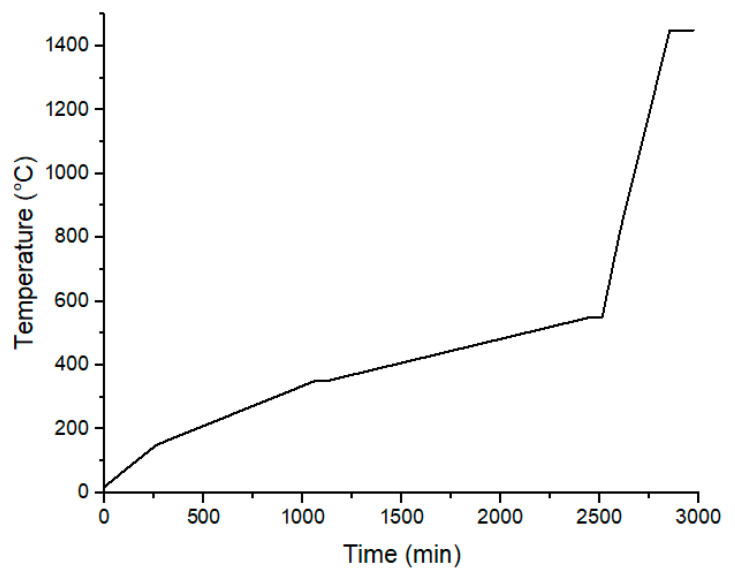
Temperature history for thermal debinding and sintering steps.

In the case of blends containing 50 vol% of Zirconia, remaining organic components were thermally debinded and the samples were sintered in an oven to produce dense ceramics. Thermal profile and procedure details are respectively shown in Figure 3 and Table 2. Those thermal procedures were developed for this particular Zirconia grade in order to favor full densification.

### 2.3. Characterization

#### 2.3.1. Rheological Study

Using a rheological model developed by Robert et al. [36] and detailed in the next section, viscosity measurements were used to investigate waxes miscibility in HDPE and PP in molten conditions. Those particular tests were realized on neat polymer/wax blends with increasing wax proportions (Table 3). Polymer and wax were mixed for 15 min at 180 °C in an internal mixer (HAAKE Rheomix 600, ThermoFisher, Waltham, MA, US). Samples of 25 mm diameter and 1 mm thick were shaped in a hot press at 180 °C. Rheological experiments in dynamic frequency sweep mode with parallel plates geometry were conducted at 180 °C using an ARES rheometer (TA Instruments, New Castle, DE, US). Before applying a 0.8 mm gap and starting the experiment, the specimens were maintained 5 min at 180 °C. From 300 to 1 rad/s the strain was set at 10% and increased to 60% at lower frequencies to maintain measurements precision.

#### 2.3.2. Scanning Electron Microscopy (SEM)

To evaluate the dispersion state of the fillers and porosity creation, scanning electron microscopy observations were realized (Quanta 250 FEG, ThermoFisher, Waltham, MA, US). After waxes extraction, 1 mm thick samples were fractured in liquid nitrogen, coated with copper, and the secondary electron signal was analyzed.

#### 2.3.3. Mercury Injection Porosimetry

After wax extraction, mercury was injected in the porous sample at high pressures with AutoPore IV 9500 porosimeter (Micromeritics, Norcross, GA, US), allowing the incremental filling of pores from 100 μm to 6 nm.

Pore size distribution was approximated from the injected volume and the increasing mercury injection pressures. In fact, the pressure needed to access a pore corresponds to the pressure needed to access the throat of the pore [37,38]:

(1) $$\begin{array}{c} D=\frac{-4\gamma cos\theta }{P} \end{array}$$

where *D* is the diameter of the pore throat, *γ* is the mercury surface tension (485.10^−2^ N.cm^−1^), *θ* is the contact angle between mercury and the sample wall (assumed at 130 °C), and *P* is the measured pressure (Pa). As the pressure is increased to enter small pores, the presence of larger pores inside the sample can be covered. Therefore, mercury porosimetry is a powerful comparative method which provides information about pore size distribution, total pore volume, and skeleton density.

#### 2.3.4. Penetration Tests

**Figure 4:**
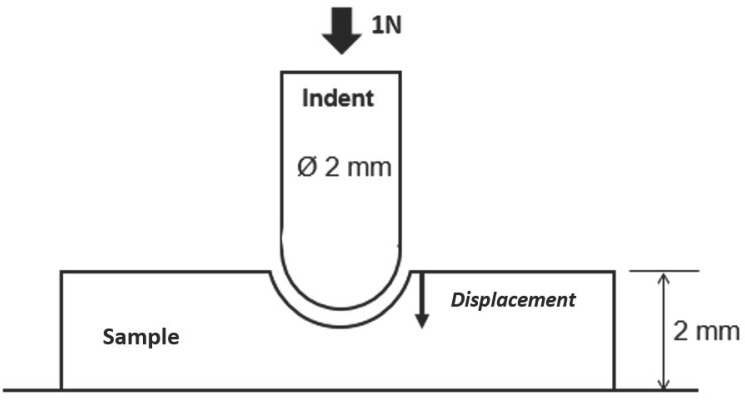
Penetration tests set-up.

Penetration tests were realized with an indent on 2 mm thick injected samples (Figure 4) using a dynamic mechanical analysis machine (DMA Q800, TA instruments, New Castle, DE, US). A constant force of 1 N and a temperature rate of 3 °C/min were applied on the sample spanning a 50 to 200 °C temperature range. By registering indent displacement as a function of the temperature, the evolution of mechanical properties of the sample is measured. Mechanical losses were observed at temperatures corresponding to the melting temperature of the organic components presenting a continuous phase in the sample. Thus, depending on the composition of the multicomponents blend, the number of mechanical losses can vary, giving an insight into phase organization.

#### 2.3.5. Density Measurements

Quality of Zirconia sintered part was evaluated by density measurements. Those tests track remaining porosity, which is mainly responsible for low mechanical performances of the part. Density tests were realized via hydrostatic measurements on a ME204 Mettler-Toledo scale (Colombus, OH, US). Samples were weighed in air and in water, and the hydrostatic density of the sample, *ρ_sample_*, was calculated based on Archimedes’ principle:

(2) $$\rho_{sample}=\frac{m_{air}}{m_{air}-m_{water}}\rho_{water}$$

where *m_air_* and *m_water_* correspond, respectively, to the sample weight in air and in water, and *ρ_water_* is the density of water at the measurement temperature. The measured ceramic densities are compared with 6.08, the maximum theoretical density of the selected Zirconia powder.

## 3. Results and Discussions

### 3.1. PP/Wax and PE/Wax Morphologies

Due to a large viscosity ratio, low molar wax addition induces a dilution effect of the backbone polymer strongly decreasing its viscosity [39], [40]. Robert et al. studied paraffin wax miscibility in both PP and HDPE at the molten state via viscoelasticity measurements [36]. They developed a model based on the comparison between polymer/wax viscosity behaviors and generalized Carreau–Yasuda equation in order to conclude on blends homogeneity.

In fact, considering an entangled regime of HDPE or PP chains, the zero shear viscosity of the homogeneous blend *η_0_* can be expressed as a function of the backbone polymer viscosity *η_0P_*, its content in the blend *φ*, and a factor relative to the free volume correction with temperature (Equation (3)) $a_{\varphi }$. The latter can be expressed as a function of the temperature, the universal gas constant, and activation energies of the blend and the polymer [36].

(3) $$\begin{array}{c} \eta_{0}=\eta_{0P}a_{\varphi }\varphi^{4}\; \end{array}$$

The influence of polymer dilution on relaxation time can be expressed by (Equation (4))

(4) $$\begin{array}{c} \tau \left(\varphi \right)=\tau_{P}a_{\varphi }\varphi^{-1.75}\; \end{array}$$

where *τ_P_* represents the relaxation time of the neat polymer. Assuming the applicability of Cox–Merz rule ($\dot{\gamma }\equiv \omega$) the viscosity of polymer/wax blends can be described by the generalized Carreau–Yasuda equation (Equation (5)), where *m* and *a* are respectively related to the power law slope and the transition between power law and zero-shear domains.

(5) η(ωφ)=η0Paφφ4[1+(τPaφφ1.75)a]ma 

By comparing PP/wax and HDPE/wax viscosity behaviors to generalized Carreau–Yasuda equation, Robert et al. concluded that PP-based blends underwent phase separation above 5 wt% of wax while HDPE-blends were homogeneous up to 30 wt% of wax, in the molten state. They also investigated the effect of crystallization on PP/wax and HDPE/wax morphology with polarized light measurements. It was shown that low molar mass component was rejected in interspherulitic regions during PP crystallization [36]. DSC measurements have also been used in the literature to investigate structure of PE/wax and PP/wax blends after crystallization [41,42].

In this study, HDPE/wax and PP/wax blends presenting weight ratios of 90/10, 80/20, and 35/65 were investigated. Based on the work of Robert et al., experimental viscosities were measured and compared to generalized Carreau–Yasuda equation by applying factors of $1/a_{\varphi }\varphi^{1.75}$ and $a_{\varphi }\varphi^{4}$ on the frequencies and viscosities, respectively [36].

**Figure 5:**
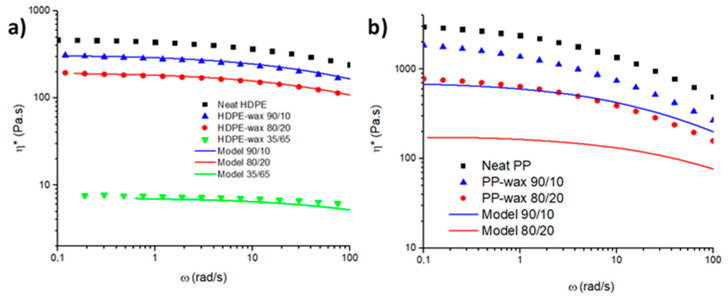
Absolute complex viscosity of polymer/waxes blends with varying wax content: (**a**) HDPE/wax and (**b**) PP/wax. The model is from the work of Robert et al. [36].

In the case of HDPE/wax blends, experimental and expected viscosities are comparable on the whole concentration range (Figure 5a). We can conclude that HDPE/wax blends form a unique and homogeneous phase. Therefore, HDPE and wax are fully miscible in the molten state.

In PP-based blends, a viscosity difference is observed even at low wax contents (Figure 5b). PP and wax present a low miscibility in the molten state, creating multiphased blends. Those results are in good agreement with the results mentioned in the literature where HDPE develops higher miscibility with waxes compared to PP [36,41,42].

**Figure 6:**
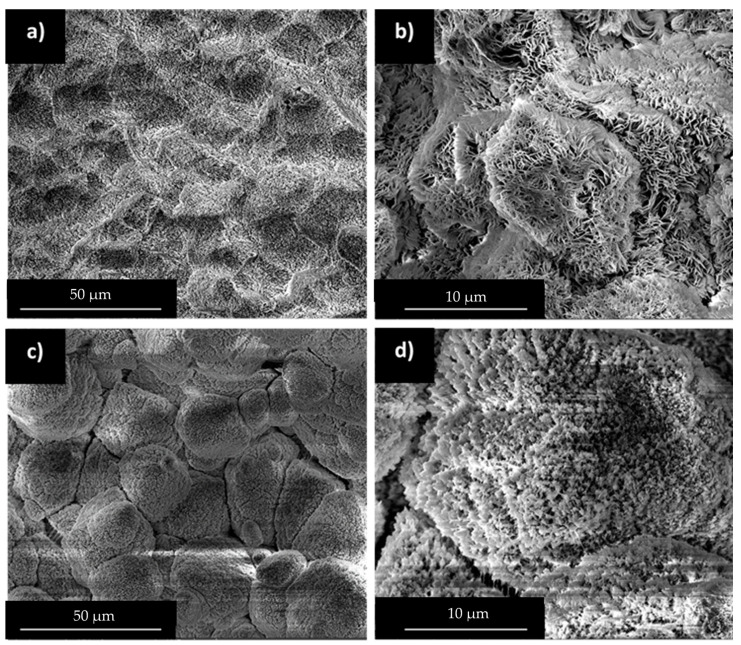
SEM observations after waxes (56 wt%) solvent extraction: (**a**) and (**b**) HDPE/wax, and (**c**) and (**d**) PP/wax.

Miscibility results at the molten state can be compared to morphology information at the solid state observed from SEM observations. HDPE/wax and PP/wax blends containing 56 wt% of wax were studied after selective wax extraction (Figure 6).

In the case of HDPE/wax blends, the surface is quite homogeneous and reveals uniform porosity surrounded by thin HDPE walls at high magnification. At the solid state, HDPE and wax form two interpenetrating phases. Despite the high miscibility of wax in HDPE in the molten state observed previously, phase separation is observed during crystallization. However, HDPE/wax high affinity favors the creation of thin domains presenting high contact area.

PP and wax form multiphased blends in the molten state and this behavior is amplified during cooling. Large cracks have been observed between PP grains at low magnification. The latter arises from wax segregation at PP grain boundaries during PP early crystallization (Table 1). At higher magnification, a thin surface roughness is detected on PP grains surface, attesting of uncompleted phase separation at the solid state. This observation can arise from partial miscibility of wax in PP in the molten state. Those results are in good accordance with Robert et al. study in which wax segregation was favored by PP crystallization [36].

**Figure 7:**
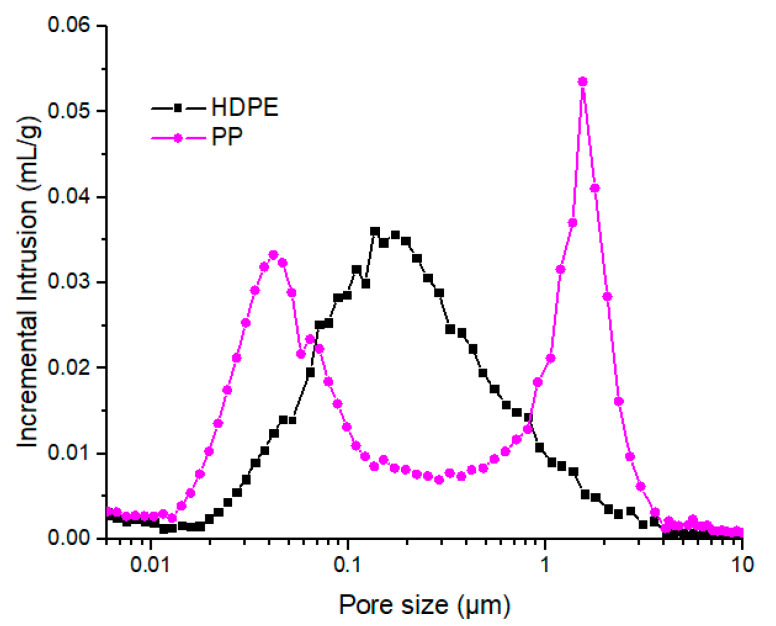
Pore size distribution after waxes (56 wt%) solvent extraction in HDPE/wax and PP/wax blends.

In addition, open-pores networks created by waxes extraction from those HDPE/wax and PP/wax blends have been characterized by mercury porosimetry (Figure 7).

HDPE/wax presented a unique large pore population centered at 200 nm. This result is in accordance with homogeneous pore structure observed in Figure 4. PP-based blends showed two different pore populations centered, respectively, at 1500 and 40 nm. Based on SEM observations, we can conclude that the large population corresponds to wax segregation at grain boundaries, while the thinnest is related to surface roughness.

In both cases, miscibility and structural conclusions are in good agreement. Wax miscibility in selected backbone polymer at high temperature highly influence matrix morphology after crystallization.

### 3.2. HDPE/PP/wax Blends Morphology

In this part, blends composed of HDPE, PP, and wax (56 wt%) with varying HDPE/PP ratios (e.g., 70/30, 50/50, and 30/70) were studied and compared to binary morphology described previously.

**Figure 8:**
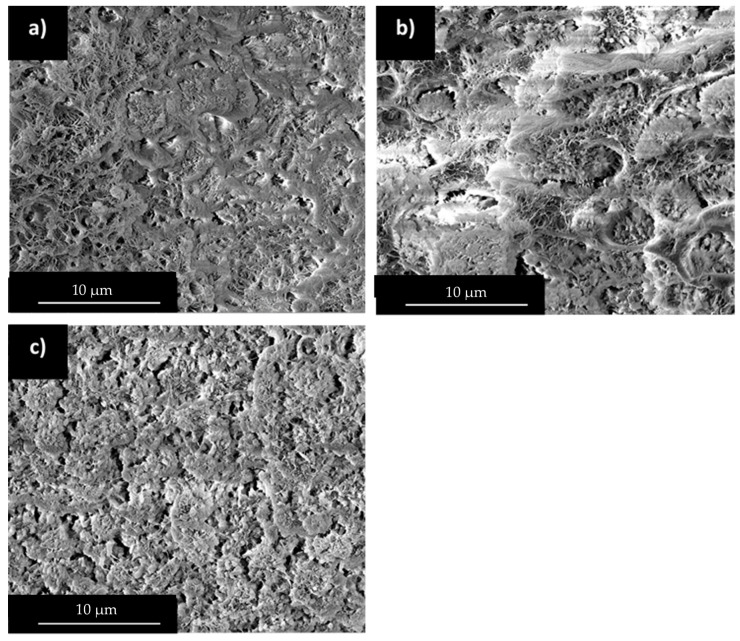
SEM observations of HDPE/PP/wax morphology after wax extraction, HDPE/PP: (**a**) 70/30, (**b**) 50/50, and (**c**) 30/70.

HDPE/PP blends have been widely studied in the literature and are known to form multiphased structures in both molten and solid states [43,44,45]. After wax extraction, SEM observations showed porous structures composed of two phases corresponding to HDPE and PP with varying proportions (Figure 8). Those phases present different pore characteristics, which are comparable to that of neat PP and HDPE in presence of wax, observed in Figure 4.

In the case of 70/30 HDPE/PP ratio, the structure is homogeneous and wax seems to be preferentially integrated in HDPE phase, due to their high affinity. As HDPE/PP ratio decreases to 30/70, HDPE phase becomes saturated in wax and cracks are progressively grown in the samples due to wax segregation.

**Figure 9:**
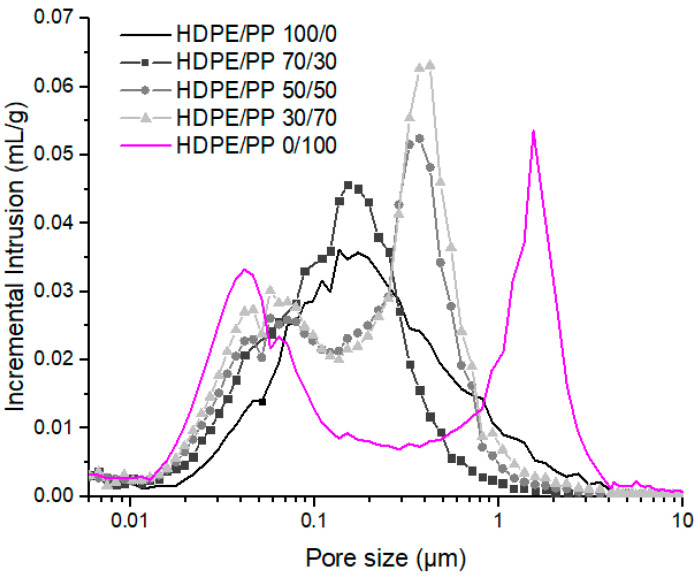
Pore size distribution from wax extraction in HDPE/PP/Wax blends.

Pore networks created during wax extraction have been analyzed via mercury porosimetry (Figure 9). For the 70/30 HDPE/PP ratio, we observe a unique pore population similar to that obtained with neat HDPE. This result is consistent with SEM observations, where wax seems mainly absorbed by HDPE phase (Figure 8a).

For both the 50/50 and 30/70 ratios, two distinct pore populations are observed. The thinnest is centered at 40 nm and its behavior presents a clear dependence on the PP content. This population seems to characterize the volume of wax dispersed in PP grain surface roughness.

The second pore population is centered at 350 nm, presenting a characteristic size slightly larger than that obtained with neat HDPE. As the HDPE/PP ratio decreases, HDPE phase becomes saturated in wax leading to an enlargement of the dispersed wax network. This observation is also consistent with SEM observations, where the apparition of small cracks for HDPE content lower than 50% has been pointed out (Figure 8b,c).

In conclusion, the selection of backbone binder and its interaction with wax has a critical impact on the structure of the organic matrix. To avoid crack development, due to wax segregation in the matrix, a HDPE/PP ratio of 70/30 or higher should be employed.

### 3.3. Zirconia Addition in HDPE/PP/Wax

In this part, zirconia is added from 0 to 40 vol% in HDPE/PP/wax (70/30) blends. At such HDPE/PP ratios, previous results highlighted that wax is mainly dispersed in the HDPE phase, in which it is highly miscible.

Using the model developed by Shivashakar et al. describing a suspension of monosized spheres, interparticle distance *δ* can be evaluated as a function of the solid loading *ϕ,* the maximum packing fraction of the fillers, and their diameter *D* (Equation (6)) [46].

(6) $$\begin{array}{c} \delta =D\left[{\left(\frac{1}{3.\pi .\Phi }+\frac{5}{6}\right)}^{0.5}-1\right] \end{array}$$

Considering random close packed monomodal spheres presenting a maximum packing fraction of 0.64, theoretical interspacing parameter can be calculated as a function of solid loading (Table 4) [47].

**Figure 10:**
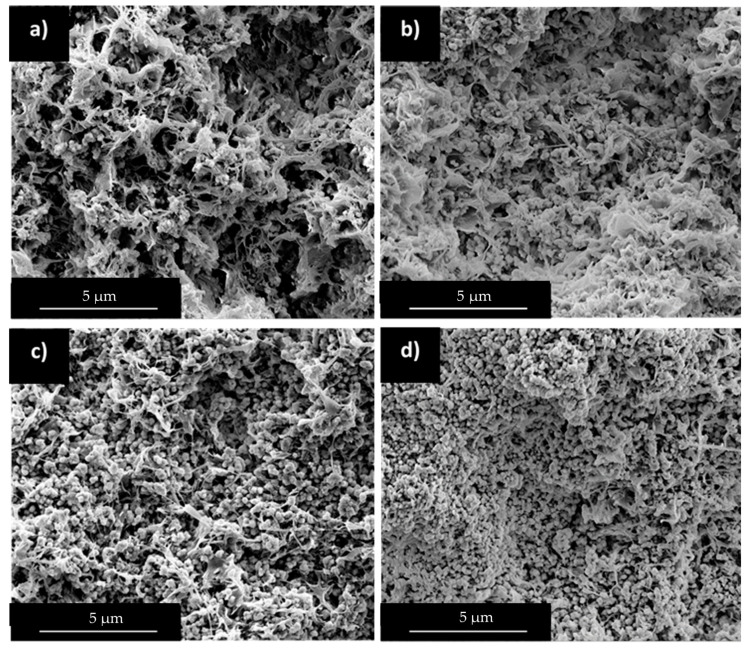
SEM observations after waxes solvent extraction (HDPE/PP ratio of 70/30), with increasing solid loadings of zirconia particles: (**a**) 10 vol%, (**b**) 20 vol%, (**c**) 30 vol%, and (**d**) 40 vol%.

SEM observations were employed after wax extraction to identify morphology evolution with increasing solid contents (Figure 10). At low zirconia contents, clusters of zirconia particles with sizes ranging from 300 nm to 600 nm are dispersed in the organic matrix, leading to slight modifications in the matrix morphology (Figure 10a). As the solid loading increases, cluster sizes expand and organic domain sizes are restricted, leading to a decrease of the observed pore size. The latter, created by waxes extraction are easily identified in black on SEM images (Figure 10b,c).

Above 40 vol% of zirconia, fillers seem to form a percolating phase in the blends, leading to a further decrease in organic domain sizes. The structure and pores become more homogeneous (Figure 10d) [6].

**Figure 11:**
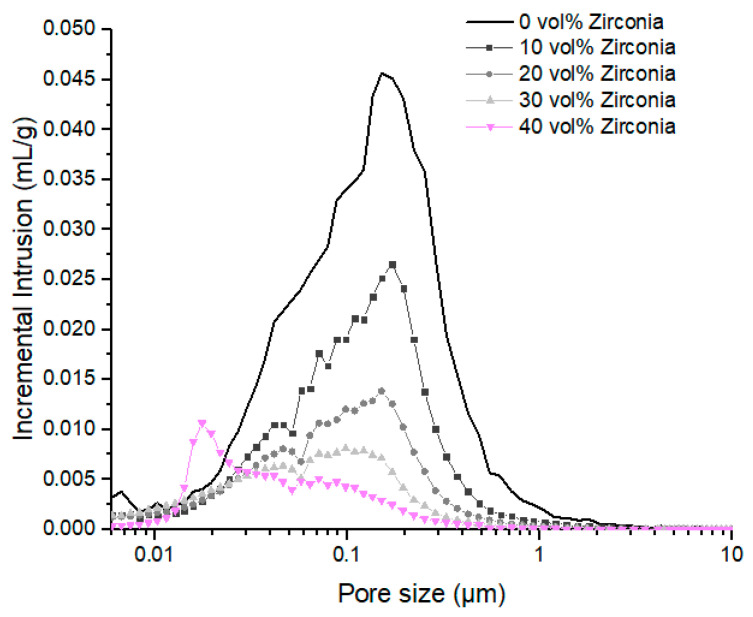
Pore size distribution after waxes solvent extraction of HDPE/PP/wax (70/30) blends for different zirconia particle concentrations.

Wax network evolution has also been characterized by mercury porosimetry (Figure 11). Under 20 vol% of zirconia, the pore size distribution is barely modified by the addition of zirconia, presenting a large population centered at 200 nm.

Between 20 vol% and 40 vol% of zirconia, the organic matrix is gradually confined between zirconia clusters, leading to a shift of the main pore population towards smaller pore sizes. In fact, above 20 vol% of zirconia, the theoretical interspacing parameter becomes lower than 200 nm, corresponding to the main pore size in the neat organic matrix. As a result, a secondary pore population is developed around 45 nm. This pore diameter is similar to the characteristic size of wax network dispersed in PP surface roughness. As the solid loading increases, the behavior of wax dispersed in HDPE phase becomes restricted, favoring the dispersion of wax at the surface of PP grains.

For solid contents above 40 vol%, interparticle distances are expected to be lower than 100 nm. Particles are closed-packed and their interparticle distances are highly restricted [48]. As a result, the main pore population previously observed disappears and a new sharp pore population centered on 20 nm is created. The phenomenon can be linked to percolation phenomenon observed on SEM results. As the matrix behavior is highly restricted and this leads to smaller and sharper pore size distributions [5,18].

Liu and Tseng studied the effect of solid content on the developed pore network after complete binder removal. Depending on ceramic powder characteristics and debinding parameters, solid contents between 45 vol% and 70 vol% were studied [5,6,18,49]. As these contents are higher than fillers percolation threshold, sharp pore populations were observed, presenting characteristic size shifted towards smaller pores as the solid content increased.

**Figure 12:**
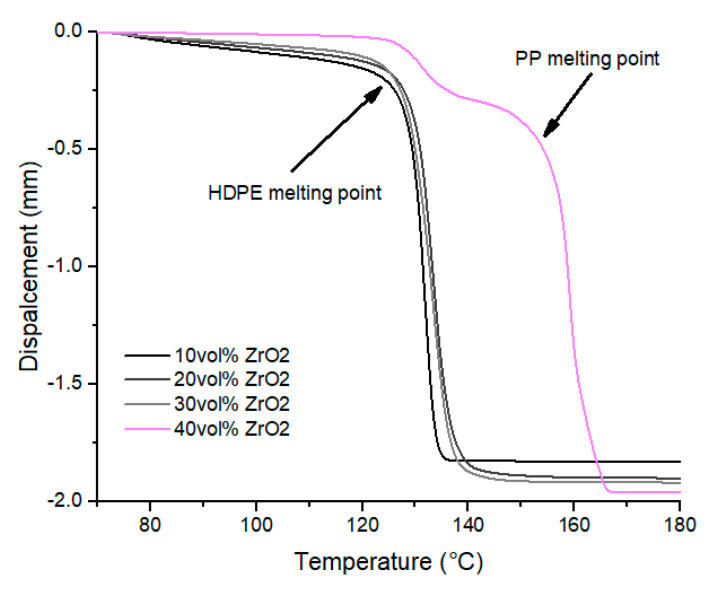
Penetration tests on blends HDPE/PP/wax (70/30) for different Zirconia particle contents.

In addition, penetration tests were used to investigate mechanical loss as a function of the melting temperature of the binders. Those observations allow us to identify the continuous phases and understand organic binder morphology and its modification with zirconia addition (Figure 12).

Under 40 vol% of zirconia, a unique and critical loss of mechanical performance is recorded around 130 °C, corresponding to HDPE melting temperature. HDPE phase, in which waxes are mainly dispersed, leads mechanical performances of the blends and constitutes the matrix, while PP forms a dispersed phase.

Beyond 40 vol% of fillers, a second step is observed at 160 °C, corresponding to PP melting temperature. Zirconia particles are close-packed leading to a modification of organic matrix structure, in which HDPE and PP phases become co-continuous.

### 3.4. Zirconia Addition with Varying HDPE/PP Ratios

**Figure 13:**
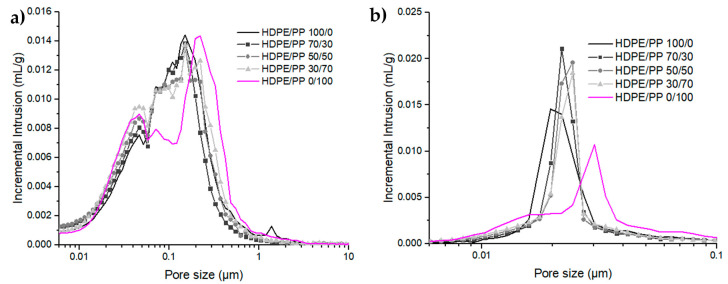
Pore size distribution after waxes solvent extraction for different HDPE/PP ratios: (**a**) 20 vol% and (**b**) 50 vol% of zirconia particles.

In this subsection, 20 vol% or 50 vol% Zirconia fillers were added to HDPE/PP/wax blends presenting different backbone binder compositions. Those samples were submitted to mercury intrusion analysis after wax extraction (Figure 13). Those results are compared to behaviors of neat organic matrices with varying HDPE/PP ratios (Figure 10). Without zirconia, two distinct pore populations were observed: the first one was highly influenced by PP content and centered at 40 nm, and the second was shifted from 1500 nm to 200 nm as HDPE/PP ratio is increased from 0/100 to 100/0. Those populations have been related to wax dispersed, respectively, in PP and HDPE phases.

At 20 vol% zirconia addition, pore size distributions are also composed of two populations. The thinnest is centered at 40 nm, regardless of HDPE/PP ratio, and is enhanced by PP increasing content. This behavior is similar to the thinnest population observed on neat polymer matrices. Thus, this population mainly characterizes the portion of wax dispersed in PP surface roughness.

The second population is centered at 150 nm for HPDE/PP ratios above 30/70, and is enlarged to 220 nm in the case of neat PP (Figure 13a). As observed in Figure 9, when the matrix is confined between zirconia clusters, the larger pore size is shifted towards smaller values, leading to smaller pores than that developed in neat matrices. At 20 vol% of Zirconia, the PP-based blend generates a second population 30% larger than that of HDPE based blends.

When 50 vol% of zirconia is added to create fully loaded feedstocks, pore distributions become monomodal and sharp due to fillers percolation and organic matrix confinement. However, as the HDPE/PP ratio is increased from 0/100 to 100/0, the pore population is shifted from 30 nm to 20 nm (Figure 13b). Similarly to blends containing 20 vol% of zirconia, the PP-based blend generates pores 30% larger than that of the HDPE-based blends.

In the literature, Wen et al. measured pore size distribution created in feedstock presenting 50 vol% solid content and a backbone binder composed of LDPE (50 vol%) and HDPE (50 vol%) after solvent and thermal debinding [34]. They observed the creation of monomodal pore distributions centered at 40 nm. This characteristic pore size is larger than that obtained in the present publication since only solvent debinding was performed.

In conclusion, backbone polymer selection has a critical impact on wax dispersion, influencing not only neat organic matrix morphology but also filled blends morphology. Even in highly confined systems such as feedstock, a modification of 30% of the pores network characteristic size is monitored between HDPE/PP ratios of 100/0 and 0/100.

Finally, samples presenting 50 vol% of zirconia particles were thermally debinded and sintered with adapted thermal procedures (Figure 3). Sintered density was measured via hydrostatic measurements in order to track remaining porosities which are highly detrimental for final ceramic parts mechanical performances (Table 5). The results showed that the highest HDPE contents lead to the highest sintered density, 6.065, corresponding to 99.7% of the maximal theoretical density of zirconia powder.

From previous results, we can assume that a higher wax miscibility in the backbone binder produces more homogeneous feedstocks, presenting thinner and sharper pores network and leading to higher sintering ability of the samples. In fact, pores network created during solvent debinding plays a critical role in both thermal debinding and sintering steps. A thin and homogeneous initial wax network reflects good fillers dispersion and general homogeneity of the whole organic matrix in the sample [6]. It favors the growth of thinner and more homogeneous pore channels after total debinding, which improves the evacuation of degradation products during thermal debinding and sintering ability [50,51], thus leading to higher final density and mechanical properties [5,49].

In the literature, Wen et al. obtained zirconia relative densities of 97.4%, 97.5%, and 98.6% for feedstocks respectively based on HDPE, PP, and HDPE/PP backbone binders [33]. They concluded that multi-polymer-based feedstocks presented higher densification ability. These lower densification abilities observed by Wen et al. can originate from either formula parameters, such as differences in wax/backbone binders affinity or processing parameters, such as the large heating rate of 5 °C/min employed during thermal debinding which can easily induce the enlargement of the pore network [6].

## 4. Conclusions

In the present publication, the use of HDPE, PP, and their blends as backbone binders in CIM feedstocks was investigated by studying the morphology of both neat organic matrices and zirconia filled blends, and the final ceramics properties.

The miscibility of paraffin wax, employed as plasticizer, with backbone binders was first studied in the molten state. The high miscibility of wax in melted HDPE hinders its segregation during crystallization, favoring the creation of thin and interpenetrating domains. The resulting pore network created after wax extraction is characterized by a unique pore population. On the contrary, wax is only partially miscible in PP in the molten state, resulting in phase segregation at high temperature. During blend crystallization, wax is mainly rejected in PP interspherulitic regions and a small proportion is dispersed in PP grains surface roughness, creating a bimodal pore population shifted toward larger pores after wax extraction.

As zirconia powder is added to create CIM feedstock, the pore size distribution resulting from wax extraction is shifted towards smaller pores. The fillers percolation is observed for solid loadings above 40 vol%, highly restricting the organic matrix organization. At such high filling ratios, the size of the pores created during wax extraction becomes sharp attesting to the homogeneity of the blend.

As the PP content in backbone binder decreases from 100 wt% to 0 wt%, the characteristic size of the pore distribution is reduced by 30%. Such refinement allows the evacuation of degradation products while limiting diffusion distances, resulting in a high ceramic densification ability. Thus, a minimum content of 70 wt% of HDPE (relative to backbone binders content) is necessary to reach the highest final zirconia density of 6.065.

The main results can be summarized as follows.

◦ A high miscibility of plasticizer in backbone binders in the molten state limits phase segregation during crystallization, thus facilitating the creation a finer and more homogeneous wax network in organic matrices.
◦ As solid content increases, interparticle distances are restricted, and the wax network is shifted towards smaller pores. Above 40 vol%, the percolation of fillers induces major modifications in the organization of organic phases and the wax network becomes monomodal and sharp, attesting to blend homogeneity.
◦ As HDPE content in backbone binder increases in 50 vol% loaded feedstocks, a thinner wax network is promoted, restricting diffusion distances and favoring high ceramic densification ability. Using a minimum HDPE content of 70 wt%, sintered densities of 6.065 are obtained.

In the future, further investigations concerning the effect of organic components structural features such as molar mass on feedstock behavior can be realized. Similar studies can also be realized in presence of other backbone polymers such as PVA, and other common plasticizers (e.g., carnauba or PE waxes).

## Figures and Tables

**Table 1:** Characteristics of binder components.

Binder	Supplier	Density	Crystallization Temperature	Degradation Temperature
Polyethylene	Axalta, Bulle, Swizterland	0.950	115 °C	460 °C
Polypropylene	Total, Paris, France	0.905	120 °C	430 °C
Paraffin wax	Alphawax, Alphen, Neetherlands	0.785	47 °C	310 °C
Stearic acid	Sigma Aldrich, Saint-Louis, MO, US	0.845	65 °C	250 °C

**Table 2:** Thermal debinding and sintering steps details.

Temperature (°C)	Speed (°C/h)	Holding Time (min)
20	0	0
150	30	0
350	15	60
550	9	60
820	180	0
1450	150	120

**Table 3:** Components (polymers and wax) concentrations of blends used for miscibility study.

HDPE (wt%)	PP (wt%)	Wax (wt%)
90	0	10
80	0	20
35	0	65
0	90	10
0	80	20

**Table 4:** Interspacing parameter values in function of solid loadings.

Solid Loading (vol%)	Interspacing Parameter (nm)
10	330
20	190
30	120
40	70
50	40

**Table 5:** Ceramic densities after sintering process of 50 vol% filled HDPE/PP/wax blends. Note that the maximal theoretical density of zirconia ceramic is 6.08.

HDPE/PP Ratio	Sintered Density
100/0	6.065
70/30	6.065
50/50	6.053
30/70	6.048
0/100	6.040
